# Modeling Drug-Carrier Interaction in the Drug Release from Nanocarriers

**DOI:** 10.1155/2011/370308

**Published:** 2011-08-10

**Authors:** Like Zeng, Lingling An, Xiaoyi Wu

**Affiliations:** ^1^Department of Aerospace and Mechanical Engineering, University of Arizona, Tucson, AZ 85721, USA; ^2^Department of Agricultural and Biosystems Engineering, University of Arizona, Tucson, AZ 85721, USA; ^3^Biomedical Engineering IDP and Bio5 Institute, University of Arizona, Tucson, AZ 85721, USA

## Abstract

Numerous nanocarriers of various compositions and geometries have been developed for the delivery and release of therapeutic and imaging agents. Due to the high specific surface areas of nanocarriers, different mechanisms such as ion pairing and hydrophobic interaction need to be explored for achieving sustained release. Recently, we developed a three-parameter model that considers reversible drug-carrier interaction and first-order drug release from liposomes. A closed-form analytical solution was obtained. Here, we further explore the ability of the model to capture the release of bioactive molecules such as drugs and growth factors from various nanocarriers. A parameter study demonstrates that the model is capable of resembling major categories of drug release kinetics. We further fit the model to 60 sets of experimental data from various drug release systems, including nanoparticles, hollow particles, fibers, and hollow fibers. Additionally, bootstrapping is used to evaluate the accuracy of parameter determination and validate the model in selected cases. The simplicity and universality of the model and the clear physical meanings of each model parameter render the model useful for the design and development of new drug delivery systems.

## 1. Introduction

Nanocarriers of various geometries and material compositions, such as liposomes, micelles, nanocapsules, polymeric nanoparticles, solid lipid particles, nanofibers, and hollow nanofibers, have been developed for the delivery and controlled release of different therapeutics [1, 2]. For instance, the use of nanoparticulate carriers has long been explored as a mechanism for delivering therapeutic and imaging agents via different administration routes, including intramuscular or subcutaneous injection, and oral and ocular administration [3]. Likewise, liposomes have successfully made their way to clinical applications [4, 5]. In contrast to the long development of nanoparticulate delivery systems, the application of fibers in drug delivery has only been intensively scrutinized in the past few years [2, 6]. Micro- and nanofibers that may mimic the structural and material characteristics of extracellular matrix are often used in tissue regeneration. Bioactive molecules such as growth factors and drugs can be incorporated into micro/nanofibers, enhancing the biochemical properties of tissue scaffolds [7] or being used as drug carriers alone [6]. 

The high surface-to-volume ratio of nanocarriers, however, presents a challenge to achieving sustained release for improving patient compliance and convenience [8]. Different mechanisms have been utilized to enhance drug-carrier interaction and drug retention over applicable time periods, such that the burst drug release may be altered or even prevented. As an example, zinc ions have been used to complex cationic peptides with the carboxyl groups presented in poly(lactide-co-glycolide) acid (PLGA) nanoparticles (NPs) [9]. Charged additives such as amines and heparins may be also included in NPs and nanofibers to retain encapsulated molecules via ionic interaction [7, 10, 11]. Still, drug-carrier interaction and subsequent drug release can be modulated by alteration in drug solubility and hydrophobicity [9, 12–14] and excipient composition and microstructure [9, 12, 13, 15–17]. Typically, drug-carrier interaction is reversible, permitting encapsulated molecules to be released in a sustained and/or controlled manner. Based on the magnitude of initial burst release and the release kinetics following the burst release, drug release profiles can be classified into four categories: high and low initial burst releases followed by little additional release and high and low initial burst releases followed by steady-state release [8]. Although a number of drug release models have been developed [18, 19], few models consider drug-carrier interactions and capture the full spectrum of drug release profiles. 

Recently, we developed a simple, three-parameter model that considers reversible drug-carrier interaction and first-order release of lipophilic drugs from liposomes, leading to a closed-form analytical solution [20]. Here, the model is used to analyze drug release from a variety of nanocarriers, including liposomes and polymeric nanocapsules, NPs, fibers, and hollow fibers. The study is focused on analyzing the influences of carrier composition (i.e., molecular weight, copolymer composition, additives) and property (i.e., pore size, hydrophobicity) and external stimuli (i.e., pH, temperature) on the release kinetics of drugs. Our goal is to reveal how carrier composition and property as well as external stimuli may modulate drug-carrier interaction and diffusion-driven release. To achieve this goal, a systematic parameter study is pursued to illustrate how each model parameter influences release kinetics. The model is then fitted to more than 60 sets of release data obtained from various delivery systems. Last, statistical analysis using bootstrapping is pursued to validate the model in selected cases. 

## 2. Theory

### 2.1. Diffusion-Driven Drug Release

**Figure 1:**
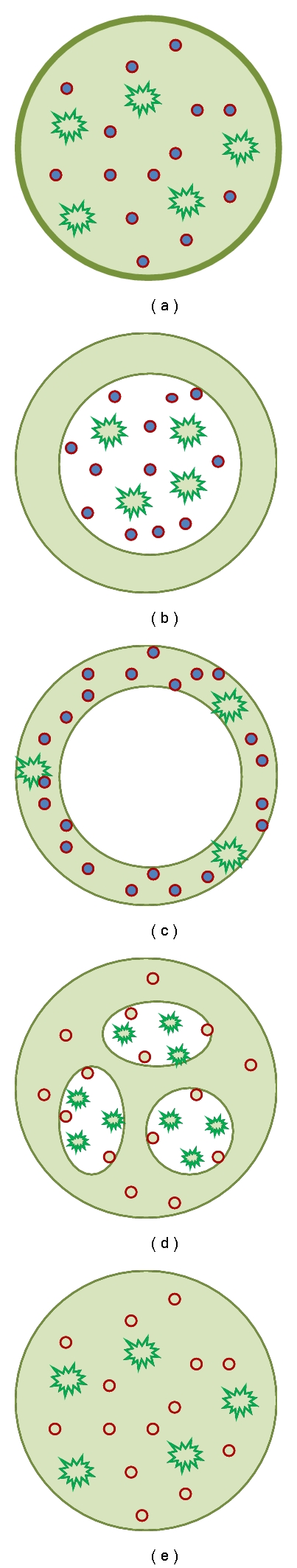
Schematics of drug release from various systems, including core-shell (a–c), porous (d), and monolithic systems (e). (a) A core functions as a drug reservoir while a shell controls release rate. (b) A special core-shell system (e.g., hollow NPs, hollow fibers, hydrophilic drugs encapsulated in liposomes), in which an inner aqueous compartment replaces the excipient in (a) as a reservoir of polymer-insoluble drug. (c) The core-shell system that encapsulates polymer-soluble drug in the shell. For instance, hydrophobic drugs are encapsulated in the lipid bilayers of liposomes. (d) A porous system with polymer-insoluble drug primarily localized in the vicinity of discrete occlusions or pores. (e) A monolithic system. Red dots represent molecularly dispersed drug molecules, while green explosions represent drug crystals/aggregates, adsorbed drug molecules on surfaces, and/or drug molecules forming complexes with the excipient. Although spherical carriers are illustrated, the schematics can apply to drug-carrier systems of fibers and other geometries.

Many drug release systems can be represented by one of the configurations illustrated in Figure 1. In this study, we consider the encapsulated drug molecules in two states: (1) the drug has been molecularly dispersed in the system and (2) drug molecules form aggregates, crystals, complexes with excipient and/or are absorbed. The latter is collectively referred as an associated drug, while the former is referred as disassociated drug molecules ready for release. Considering the reversible association/disassociation and the nonconstant concentration of a disassociated drug, the diffusion process of the molecularly dispersed drug molecules in configurations (a) and (b) in Figure 1 follows the first-order kinetics [18]:

(1) $$\frac{dm}{dt}=\frac{d\left(Vc\right)}{dt}=-Ak_{1}c\;\text{or }\frac{dc}{dt}=-k_{S}c,$$

where *t* is time, *m* and *c* are the drug amount and average drug concentration in a carrier, *V* and *A* are the volume and surface area of the carrier, and *k*_1_ is the rate constant. Here, *k*_1_ may be defined as *k*_1_ = *DK*/*l*, where *D* is the diffusion coefficient of the drug within the rate-controlling shell, *K* is the partition coefficient of the drug between the shell and the core, and *l* is the thickness of the shell [18]. The parameter *k*_*S*_ = *Ak*_1_/*V* in the rearranged form of (1) suggests that a high surface-to-volume ratio (*A*/*V*) of nanostructured carriers enhances drug release. Equation (1) was derived in perfect sink conditions, such that the drug concentration in the extracarrier medium is negligible. Additionally, this model does not consider changes in the carrier volume that may be induced by drug release and/or matrix degradation. 

In configuration (c) (Figure 1(c)), the thin membranes (e.g., the lipid bilayers of liposomes are only several nanometer thick) may render the convection of polymer-soluble drug at the carrier surface dominant. As a result, the release of drug molecules from the outer surfaces of drug carriers to the extracarrier medium follows *dm*/*dt* = −*Ah*(*c* − *c*_*∞*_) [20]. Here, *h* is the convection coefficient, which is determined by the flow characteristics of the extracarrier medium; and *c*_*∞*_ is the drug concentration in the extracarrier medium. 

In the porous and monolithic configurations (Figures 1(d) and 1(e)), transport of drug molecules in the carrier may be mediated by diffusion, excipient erosion/degradation, and/or osmotic pressure. The osmotically mediated flux of drug molecules can be written as *dm*/*dt* = − *AP*(*c* − *c*_*∞*_), where *P* is the permeability. Under perfect sink conditions, the convection-dominated and osmotic pressure-mediated release follows the first-order kinetics in (1), leading to an analytical solution of an exponential function. In contrast, a solution to diffusion-driven release in the monolithic systems is comprised of an infinite series of exponential terms [21]. Because this study focuses on the effects of drug-carrier interaction on drug release, transport of drug molecules via various mechanisms is described by the first-order kinetic model in (1). While the model provides an accurate description of several release mechanisms, it only approximates diffusion-driven release. Nevertheless, this simplification is necessary for obtaining an analytical solution when drug-carrier interaction is considered in drug release from various nanomaterials. 

### 2.2. Drug-Carrier Interaction

In addition to the transport of drug molecules, drug-carrier interaction is another important mechanism dictating the drug release profiles. Drug molecules may directly interact with drug carriers, lowering their solubility and/or retarding their release from drug carriers. Drug molecules may complex with each other or additives and then interact with drug carriers. To simplify the model, drug molecules that are not molecularly dispersed in the system are assigned collectively into a group called associated molecules, which need to be disassociated from carriers prior to release. The association and disassociation processes are assumed to be reversible. Furthermore, the reversible association of a drug molecule with a carrier is assumed to follow the first-order kinetics, in a fashion similar to reversible drug-stent interactions [22, 23]. After taking the transport process into account, the concentrations of disassociated (or “free”) and associated drugs, *c*_*F*_ and *c*_*A*_, can be obtained as follows:

(2) $$\begin{array}{c} \frac{dc_{F}}{dt}=-\left(k_{S}+k_{\mathrm{on}\;}\right)c_{F}+k_{\mathrm{off}\;}c_{A},\\ \frac{dc_{\text{A}}}{dt}=k_{\mathrm{on}\;}c_{\text{F}}-k_{\mathrm{off}\;}c_{\text{A}}, \end{array}$$

where *k*_*on*_ is the rate constant of association and *k*_*off*_ is the rate constant of disassociation. At time *t* = 0, the association and disassociation are at equilibrium, such that *c*_*F*_(0)/*c*_0_ = *k*_*off*_/(*k*_*on*_ + *k*_*off*_), *c*_*A*_(0)/*c*_0_ = *k*_*on*_/(*k*_*on*_ + *k*_*off*_), and *c*_0_ = *c*_*F*_(0) + *c*_*A*_(0) are the initial concentration of the drug. The linear system of the first-order differential equations (2) can be readily solved (see the detailed derivation in supporting information), yielding an analytical solution:

(3) $$\begin{array}{c} \frac{c_{\text{F}}\left(t\right)}{c_{0}}=\frac{k_{\mathrm{off}\;}}{k_{\mathrm{on}\;}+k_{\mathrm{off}\;}}\left[\frac{k_{\text{S}}-\lambda_{2}}{\lambda_{\text{1}}-\lambda_{2}}e^{-\lambda_{\text{1}}t}+\frac{\lambda_{\text{1}}{-k}_{\text{S}}}{\lambda_{\text{1}}-\lambda_{2}}e^{-\lambda_{\text{2}}t}\right],\\ \frac{c_{\text{A}}\left(t\right)}{c_{0}}=\frac{k_{\mathrm{on}\;}}{k_{\mathrm{on}\;}+k_{\mathrm{off}\;}}\left[\frac{-\lambda_{2}}{\lambda_{\text{1}}-\lambda_{2}}e^{-\lambda_{\text{1}}t}+\frac{\lambda_{\text{1}}}{\lambda_{\text{1}}-\lambda_{2}}e^{-\lambda_{\text{2}}t}\right], \end{array}$$

where $\lambda_{1,2}=\left[k_{\text{S}}+k_{\mathrm{on}\;}+k_{\mathrm{off}\;}\pm \sqrt{{\left(k_{\text{S}}+k_{\mathrm{on}\;}+k_{\mathrm{off}\;}\right)}^{2}-4k_{\text{S}}k_{\mathrm{off}\;}}\right]/2$, and −*λ*_1_ and −*λ*_2_ are eigenvalues of the linear system of equations (2). The cumulative drug release *M*_*t*_ = *V*(*c*_0_ − *c*_*F*_ − *c*_*A*_) can be normalized by the initial amount of drug (*M*_0_ = *Vc*_0_), leading to

(4) $$\begin{array}{cc} \frac{M_{t}}{M_{0}} & =\frac{\lambda_{2}\left(k_{\text{S}}-\lambda_{2}\right)}{\left(k_{\mathrm{on}\;}+k_{\mathrm{off}\;}\right)\left(\lambda_{\text{1}}-\lambda_{2}\right)}\left(1-e^{-\lambda_{\text{1}}t}\right)\\ & \;+\frac{\lambda_{1}\left({\lambda_{1}-k}_{\text{S}}\right)}{\left(k_{\mathrm{on}\;}+k_{\mathrm{off}\;}\right)\left(\lambda_{\text{1}}-\lambda_{2}\right)}\left(1-e^{-\lambda_{\text{2}}t}\right). \end{array}$$

Equation (4) shows that drug release profiles are determined by two exponential functions. Indeed, the model considers first-order diffusion/convection and drug association/disassociation. It is anticipated that the two mechanisms would lead to two exponential release modes. The analytical solution also reveals the full coupling of the two mechanisms. 

To further illustrate the physical meaning of the analytical solution, we consider two special cases. Case  1 corresponds to the fast disassociation of drug molecules from the carrier such that *k*_*on*_ ≫ *k*_*off*_. As a result, most of the drug molecules are initially free, and the drug release profiles are determined by diffusion and convection only. The solution in (4) is reduced to

(5) $$\frac{M_{t}}{M_{0}}=1-e^{-k_{\text{S}}t}.$$

Case  2 corresponds to fast diffusion/convection but slow association/disassociation such that *k*_*S*_ ≫ *k*_*on*_ and *k*_*S*_ ≫ *k*_*off*_. This leads to a decoupling of drug association/disassociation from drug diffusion/convection: the fast release of initially free drug molecules via diffusion/convection and the slow release of initially bound drug molecules that is dictated by the disassociation process. Accordingly, the solution in (4) is reduced to

(6) $$\frac{M_{t}}{M_{0}}=\frac{k_{\mathrm{off}\;}}{k_{\mathrm{on}\;}+k_{\mathrm{off}\;}}\left(1-e^{-k_{\text{S}}t}\right)+\frac{k_{\mathrm{on}\;}}{k_{\mathrm{on}\;}+k_{\mathrm{off}\;}}\left(1-e^{-k_{\mathrm{off}\;}t}\right).$$

The free energy difference between the free and bound states, Δ*G* = −*k*_*B*_ *T*ln (*k*_*on*_/*k*_*off*_), determines the amounts of initially free and bound drug. Here, *k*_B_ is the Boltzmann's constant, and *T* is the absolute temperature (assumed to be 300 K). In this study, therefore, three parameters, Δ*G* (instead of *k*_*on*_), *k*_*S*_, *k*_*off*_, are used to describe the cumulative drug release obtained in (4).

### 2.3. Parameter Study

**Figure 2:**
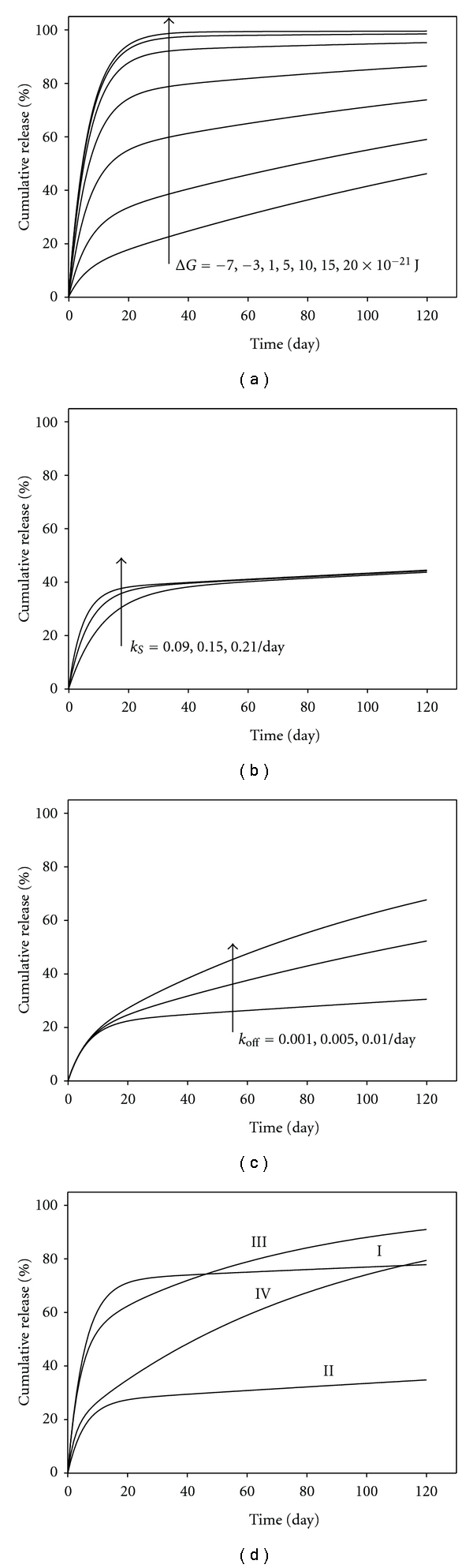
Dependence of release kinetics on model parameters. (a) Free energy difference Δ*G* (*k*_*S*_ = 0.15, *k*_*off*_ = 0.005 day^−1^). (b) Diffusion/convection rate constant *k*_*S*_ (Δ*G* = −2 × 10^−21^ J) *k*_*off*_ = 0.001 day^−1^). (c) Disassociation rate constant *k*_*off*_ (Δ*G* = −2 × 10^−21^ J, *k*_*S*_ = 0.15 day^−1^). (d) The model resembles the four categories of drug release profiles. Δ*G*, *k*_*S*_, *k*_*off*_ used in (4): 4 × 10^−21^ J, 0.18, 0.002 day^−1^ for category I; −4 × 10^−21^ J, 0.18, 0.001 day^−1^ for category II; 1 × 10^−21^ J, 0.24, 0.015 day^−1^ for category III; and −5 × 10^−21^ J, 0.36, and 0.013 day^−1^ for category IV). In (a)–(c), solid and dashed lines represent the model prediction using (4) and (6), respectively.

A parameter study based on (4) reveals the significant influence of Δ*G* on the magnitude of initial burst release (Figure 2(a)). If Δ*G* is comparable to *k*_*B*_ *T* (*≈*4.14 × 10^−21^ J), more than 70% of the drug will be released during the phase of initial burst release. Lowering Δ*G* promotes the drug-carrier association, reducing initial burst release and enhancing steady release. The rate constant of diffusion/convection affects the rates, but not magnitude, of the initial burst release (Figure 2(b)). Likewise, increasing *k*_*off*_ enhances steady release following initial burst release (Figure 2(c)) but has no effect on the magnitude of initial burst release. For comparison, the model prediction using (6) is also presented in Figures 2(a)–2(c). Consistent with our theoretical analysis, (4) and (6) yield nearly identical results, if *k*_*S*_ ≫ *k*_*on*_ and *k*_*S*_ ≫ *k*_*off*_. However, if the conditions (*k*_*S*_ ≫ *k*_*on*_  and  *k*_*S*_ ≫ *k*_*off*_) are not satisfied, (6) will lead to a higher prediction of cumulative release than (4), because diffusion and convection are neglected during the steady-state release phase in (6). Interestingly, this simple model is capable of replicating the four categories of drug release profiles that were classified by Ye et al. [8]: high initial burst release with little additional release (I), low initial burst release with little additional release (II), high initial burst release with steady-state release (III), and low initial burst release with steady-state release (IV). 

## 3. Results and Discussion

**Figure 3:**
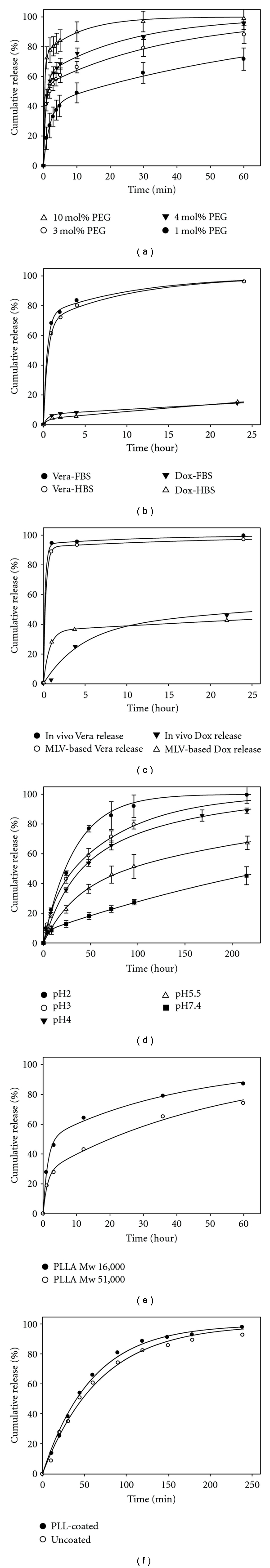
The model fit into release data. (a) Carboxylfluorescein from thermosensitive liposomes with different PEG addition [24]. (b, c) Verapamil and doxorubicin from liposomes [25]. (d) Amiodarone from LNC [26]. (e) BSA from PLLA nanocapsules [29]. (f) Indomethacin from PECL nanocapsules [27]. Solid lines represent the model prediction.

**Figure 4:**
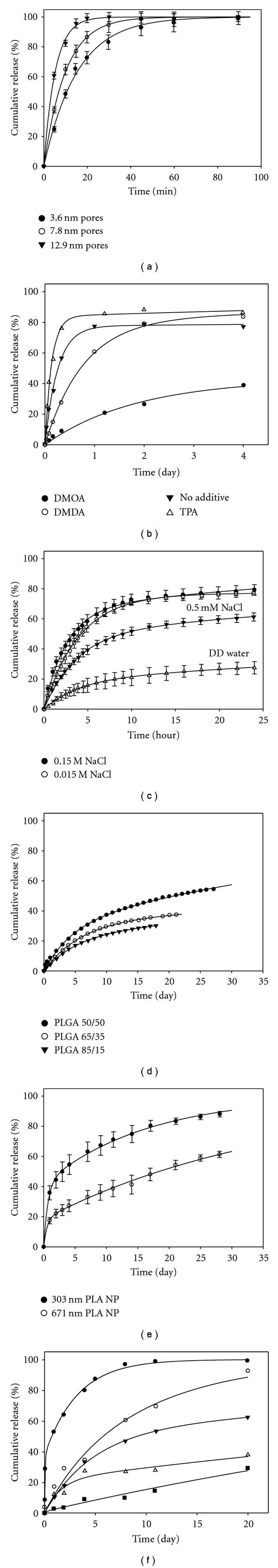
The model fit to release data. (a) Telmisartan from MSNPs with different pore sizes [30]. (b) Synthetic retinoid Am80 from PEG-PBLA NPs with different amine additives [11]. (c) DS from PLNPs in the release medium of various ionic strengths [10]. (d) Estradiol from PLGA NPs [12]. (e) Savoxepine from PLA NPs of two different sizes [13]. (f) Steroids from various PLA/PLGA NPs (■: PLA Mw 14,000; ∆ PLA Mw 9,000; *▾*: PLGA Mw 13,000; ○: PLGA Mw 8,000). BDP release (●) from PLA NPs (Mw 14,000) is a control [9]. Solid lines represent the model prediction.

**Figure 5:**
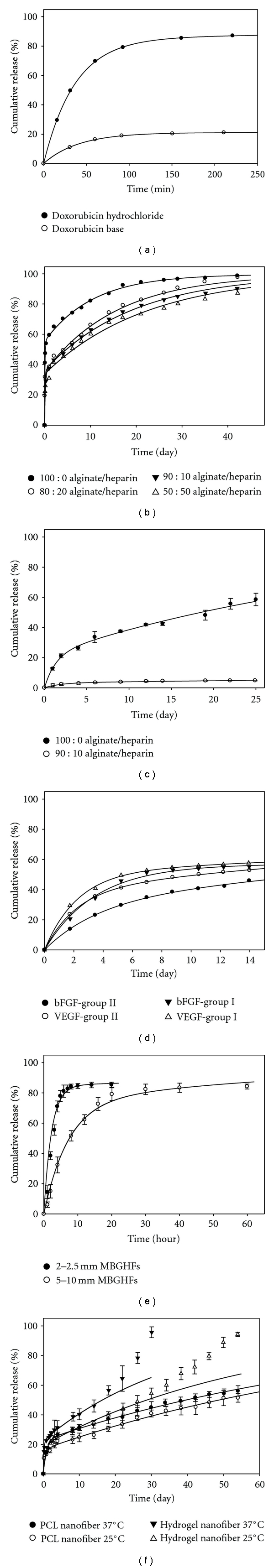
The model fit to release data. (a) Doxorubicin release from PLLA NFs [14]. (b) Avidin. (c) PDGF from alginate/heparin composite fibers [7]. (d) VEGF and bFGF from PEtU-PDMS/fibrin composite fibers [15]. (e) GS from MBGHFs with different lengths [16]. (f) BSA release from hydrogel and PCL nanofibers [31]. Solid lines represent the model prediction.

To test the model, we fit it to 60 sets of release data from 16 carrier systems, which include liposomes and nanocapsules (Figures 3(a)–3(f)), nanoparticles (Figures 4(a)–4(f)), and nanofibers (Figures 5(a)–5(f)). The release data were collected in nearly perfect sink conditions. To obtain the release profiles of drug, a small volume of drug-loaded carriers may be added into a large volume of release medium either directly [24] or indirectly via a dialysis bag [25–27]. Release kinetics of these drug-carrier systems covers all four categories illustrated in Figure 2(d). Because some release data include the mean and standard variation, but others are simply representative cumulative release values, in this study, we fit the model to the mean or representative release curves only.

### 3.1. Parameter Determination

Because each model parameter has clear physical meaning, a simple method has been developed to estimate the model parameters (see supporting information). Briefly, we use the estimated magnitude of the initial burst release to evaluate Δ*G*. Next, the initial release rate (at *t* = 0) is used to estimate *k*_*S*_. Last, *k*_*off*_ that determines the kinetics of the sustained release is calculated. These estimated parameters (i.e., Δ*G*, *k*_*S*_, *k*_*off*_) are used as the initial input in Matlab codes to refine the estimations using an optimization method. The properties of the parameter estimates, such as mean and standard deviation, are assessed using bootstrap sampling [28], as detailed in Section 3.5. 

### 3.2. Drug Release from Liposomes and Nanocapsules

Liposomes and lipid nanocapsules (LNC) are among drug delivery systems that first made their way to clinical applications [5]. The bilayered structure of liposomes enables the encapsulation of hydrophilic and lipophilic drug in their interior aqueous compartments (Figure 1(b)) and in the lipid bilayers (Figure 1(c)), respectively [32]. However, liposomes can be easily trapped by the reticuloendothelial system (RES), leading to rapid removal from circulation [33]. A hydrophilic barrier, often formed by polyethylene glycol (PEG) derivatives, may be created to protect liposomes, avoiding their uptake by RES [34]. PEGylation of liposomes increased their circulation half-times of about 30 minutes to 5 hours nearly two decades ago [34] to around 10 hours recently [35], enhancing their spontaneous accumulation in solid tumors [34, 36]. Efforts to control release kinetics made it possible to deplete encapsulated drugs in a time comparable to or longer than the circulation time of liposomes [25, 26]. Here, we simulate drug release from liposomes and LNC at different time scales (Figures 3(a)–3(d)) and from polymeric nanocapsules (NC) for comparison (Figures 3(e) and 3(f)). Parameter estimates for the simulations are listed in Table 1. 

We first simulate the fast release of CF from TSL, triggered by mild hyperthermia (Figure 3(a)). Li et al. [24] designed and synthesized TSL such that its gel-to-liquid transition temperature resided at around 43°C. As a result, TSL was stable at 37°C and capable of retaining encapsulated molecules in the circulation. Once it reached the targeted site, TSL released encapsulated molecules rapidly due to the gel-to-liquid transition induced by local hyperthermia. This process can be modulated by PEG addition. For instance, TSL with a high PEG density releases CF faster than TSL with a low PEG density. Our model successfully captures CF release from TSL with different PEG densities at 42°C. In particular, both Δ*G* and *k*_*S*_ increase with PEG density, suggesting that PEGylation not only modifies the permeability of the lipid membrane but also decreases the ability of TSL to interact with hydrophilic molecules. This is consistent with the report [24] that PEG at a high density destabilizes the lipid membrane of TSL and changes the membrane modality for CF release.

Next, we model the release of hydrophilic doxorubicin hydrochloride and hydrophobic verapamil from liposomes (Figure 3(b)). It has long been observed that liposomes release encapsulated molecules much faster in vivo than in vitro [25]. A speculation is that protein and lipid constituents in the in vivo environment may provide additional driving forces for release of encapsulated molecules. Indeed, serum addition slightly increases the rates of initial burst release of both doxorubicin and verapamil. The model reveals that, upon serum addition, *k*_*S*_ and *k*_*off*_ remain nearly the same but Δ*G* increases. Likely, serum addition changes the drug-carrier interactions and slightly enhances the drug release. However, serum addition alone cannot explain discrepancies between in vivo and in vitro release data. Shabbits et al. [25] proposed that the vast lipid membrane pool existing in the physiological setting induced fast release of encapsulated molecules and that inclusion of excessive multilamellar vesicles (MLV) in an in vitro assay may improve the prediction of the in vivo performance of liposomes. We fit the model to release data obtained from an in vivo study and the MLV-based assay. Interestingly, the inclusion of excessive MLV induces appreciable increases in Δ*G*, but modest changes in *k*_*off*_ (Figure 3(c)). As a result, Δ*G* obtained using the MLV-based assay is more comparable to that obtained from the in vivo study. Although the underlying mechanisms remain poorly understood, our model study suggests that the existence of lipid constituents alters the interactions between drugs and liposomes.

We also simulate the pH-dependent release of amiodarone from LNC, which possesses better stability than liposomes (Figure 3(d)). Using the MLV-based assay, Lamprecht et al. [26] examined increasing solubility and release rates of amiodarone from LNC, when pH decreased. Amiodarone displayed nearly negligible solubility at pH 7.4 but was highly soluble at pH 2.0. As a result, amiodarone release is well described by a single exponential function (5) at pH 2.0, indicating the inability of LNC to interact with and retain amiodarone in highly acidic conditions. After a 5% initial burst release, a nearly zero-order release of amiodarone was observed at pH 7.4 over a time period of 200 hours. The low burst release is likely due to an immediate dissolution of a small amount of adsorbed drug on the LNC surface. Indeed, Δ*G* of −9.3 × 10^−21^ J indicates a tiny amount of free drug available for initial burst release. The strong pH effects on amiodarone release are further revealed by the modeling study of the release at intermediate pH values. Specifically, Δ*G* decreases from 4.52 × 10^−21^ J at pH 3.0 to 3.49 × 10^−21^ J at pH 4.0 and to −0.86 × 10^−21 ^J at pH 5.5. When pH increases from 3.0 to 5.5, *k*_*off*_ also slightly decreases from 0.01 hour^−1^ to 0.004 hour^−1^. In contrast, the model parameter *k*_*S*_ remains nearly unchanged at pH from 2.0 to 5.5. The model thus suggests enhanced amiodarone-LNC interactions and thus decreased association of amiodarone at high pH. 

Like liposomes and LNC, polymeric NCs have been also explored for drug release (Figures 3(e) and 3(f)). Lu et al. [29] prepared PLLA NCs without stabilizer and analyzed the release of BSA from PLLA NCs. When PLLA with molecular weights of 16 and 51 kD is used in the preparation of NCs, the model reveals that *k*_*S*_ and *k*_*off*_ remain nearly unchanged. However, Δ*G* decreases from 0.41 to −3.3 × 10^−21^ J, suggesting that high molecular weight PLLA enhances BSA-excipient interactions and thus the entrapment of BSA molecules in the carrier. Consistent with the fact that the two types of PLLA NCs release BSA at a comparable rate in the steady-state release phase, an increase in the molecular weights of PLLA induces slight changes in the rate constants of disassociation. Beside particle size and excipient composition, the surface charge of carriers can profoundly influence the in vivo delivery and accumulation of drug at the site of action. Calvo et al. [27] reported that the coating of PECL NCs using the cationic PLL significantly improves the corneal penetration of indomethacin and thus its ocular bioavailability. Moreover, the PLL coating does not alter the release profiles of indomethacin. Indeed, the simulation shows slight or little change in all three model parameters. 

### 3.3. Drug Release from Nanoparticles

Compared to liposomes, NPs may possess improved stability. Nevertheless, various mechanisms need to be explored for enhancing NP-drug interaction and achieving sustained release. For instance, NPs prepared from poly(lactic acid) (PLA), poly(glycolic acid) (PGA), and PLGA may release hydrophobic drug in a sustained manner, due to the strong hydrophobic interaction between NPs and drug molecules [12, 13]. The sustained release of the encapsulated drug may be regulated by matrix degradation, which, in turn, can be adjusted by changing the lactide/glycolide ratio and molecular weight [9, 12]. To encapsulate a hydrophilic drug, additives capable of converting hydrophilic molecules into hydrophobic ones via ion pairing can be included [9]. Additives such as metal ions and charged polymers may form complexes with drug molecules and/or NPs [9–11]. As a result, the ionic strength of the release medium may potentially affect release kinetics of an encapsulated drug [10]. In this study (Figures 4(b)–4(f)), we use the model to analyze the influences of charged additives [11], the release medium [10], the matrix composition and molecular weight [9, 12], and the particle size [13] on release profiles of various drugs from NPs. For comparison, the rapid release of telmisartan (TEL) from mesoporous silica nanoparticles (MSNPs), in which none of the mechanisms given above is explored [30], is also simulated (Figure 4(a)). Parameter estimates for the simulations are listed in Table 2. 

We first analyze the rapid release of TEL, an angiotensin II receptor antagonist for treating hypertension, from MSNPs with different pore sizes (Figure 4(a)). TEL molecules are capable of forming weak hydrogen bonds with the silanol groups on the pore walls of MSNPs [30]. However, entropy loss associated with the formation of hydrogen bonds may make TEL less energetically favorable to complex with MSNPs. Therefore, TEL release from MSNPs may correspond to Case I (5). Indeed, only a single parameter, *k*_*S*_, is needed for describing TEL release. Moreover, *k*_*S*_ decreases as the pore size decreases, suggesting that smaller pores reduce diffusivity and TEL release rates. In contrast to the complete initial burst release of TEL from MSNPs within 80 minutes, a steady release following the 40% burst release is achieved by functionalizing MSNs using aminopropyl groups to create AP-MSNs. As a result, the three-parameter model is needed for capturing the biphasic release profiles of TEL-AP-MSNPs, in which Δ*G* is −1.2 × 10^−21^ J (see Figure  S1 in supplementary material available online at doi:10.1155/2011/370308). This is consistent with the carboxyl groups of TEL that are capable of strongly interacting with the amines of AP-MSNPs rather than the hydroxyl groups of nonfunctionalized MSNPs. 

Next, we simulate the release of synthetic retinoid Am80 from PEG-PBLA micelles (Figure 4(b)). Am80 displays rapid release in Dulbecco's phosphate buffered saline (D-PBS), due to its high solubility that is attributed to the hydrophilic carboxylic groups [11]. In order to achieve sustained release, amines capable of ion pairing with the carboxylic groups of Am80 are added into PEG-PBLA micelles. The model successfully captures the influences of different amines on the retardation of Am80 release. In particular, addition of DMDA greatly reduces burst release, leading to sustained release. The model reveals a decrease in *k*_*S*_ (from 3.91 to 1.27 day^−1^), which is responsible for the prolonged initial burst release. Likely, the Am80-DMDA pairs possess a lower diffusivity than Am80 does in PEG-PBLA micelles. Additionally, increases in *k*_*off*_ (from 0.01 to 0.06 day^−1^) and in Δ*G* (from 5.1 to 6.6 × 10^−21^ J) suggest a weaker interaction between Am80-DMDA pairs and PEG-PBLA micelles. As a result, Am80 release from DMDA-included PEG-PBLA micelles surpasses that from micelles without additive. Inclusion of DMOA has more pronounced effects on retarding Am80 release. Indeed, *k*_*S*_ decreases from 3.91 to 0.54 day^−1^, and Δ*G* decreases from 5.1 to −1.2 × 10^−21^ J. Compared to DMDA, DMOA has 12 more methylene groups. It is likely that the increased number of methylene groups not only increases the hydrophobicity and lowers the diffusivity of Am80-DMOA but also enables Am80-DMOA pairs to hydrophobically interact with PEG-PBLA micelles, leading to a more sustained release of Am80. In marked contrast, an addition of triphenylamine increases both the magnitude and rate of initial burst release. An increase in *k*_*S*_ and Δ*G* indicates that inclusion of triphenylamine not only increases Am80 diffusivity but also enables the dissociation of Am80 from the drug carriers. 

Ion pairing between drug and carrier can be influenced by the ionic strength of release medium. Li et al. [10] examined the effects of release medium on DS release from PLNPs. In their study, verapamil hydrochloride (VRP) was added into PLNPs to form a complex with DS, and the VRP-DS complex interacted with PLNPs. It was anticipated that counter ions in the release medium may interact with the sulfate groups on DS and alter DS-VRP complexes, affecting DS release kinetics of PLNPs. Indeed, when the ionic strength of the release medium increased from 0 to 0.15 M NaCl, the release rates of DS increased significantly (Figure 4(c)). Our simulations show that Δ*G* increases from −5.1 × 10^−21^ J in DDI water to 0.64 × 10^−21^ J in 0.5 mM NaCl and to 4.9 × 10^−21^ J in 0.015 M NaCl and 3.36 × 10^−21^ J in 0.15 M NaCl. The rate constant of dissociation also steadily increases from 0.005/hr in DDI water to 0.023/hr in 0.15 M NaCl. In contrast, no significant changes in *k*_*S*_ are observed. Therefore, the model suggests that the ionic strength of the release medium strongly affects the DS-VRP-PLNP association and disassociation, but not the DS diffusivity in PLNP. 

The chemical composition of NPs is another important determinant of release kinetics. Mittal et al. [12] analyzed the composition influence on estradiol release from PLGA NPs. In general, release may be mediated through both drug diffusion and matrix degradation. When high molecular weight PLGA was used to prepare NPs, however, release was largely mediated through the diffusion process. Furthermore, increasing lactide content reduced release rate (Figure 4(d)). When the PLA/PGA ratio increases from 50 : 50 to 65 : 35 and to 85 : 15, Δ*G* decreases from −1.7 to −2.4 and to −3.9 × 10^−21^ J, explaining the decreasing magnitude of initial burst release. Negative Δ*G* in all cases suggests a strong interaction between estradiol and PLGA, responsible for sustained release. In addition, a reduction in *k*_*off*_ (from 0.02 to 0.004 and 0.007 day^−1^) is consistent with the observed decrease in steady release. 

Particle size also strongly influences drug release through mediating both diffusion and matrix degradation. As shown in Figure 4(e), it takes 3 and 18 days to release 50% savoxepine from PLA NPs of 303 nm and 671 nm in size, respectively [13]. In (1), which describes the diffusion and convection process, *k*_*S*_ is proportional to the surface-to-volume ratio (*A*/*V* ∝ 1/*r*), if the rate constant *k*_1_ is independent of particle size *r*. Therefore, if release is dominated by the diffusion/convection process, doubling particle size will double the time for releasing drug at the same percentage. Thus, the diffusion process alone cannot explain the size effects observed in savoxepine release from PLA NPs. The simulation reveals a comparable *k*_*S*_ (1.44 versus 1.79 day^−1^) in both cases. In contrast, a large difference in Δ*G* (−0.61 versus −5.52 × 10^−21 ^J) suggests a stronger interaction between savoxepine and larger PLA NPs. A possible explanation is that the release medium-mediated matrix degradation, which facilitates drug-carrier disassociation, is slower in large NPs. 

The combined effects of ion pairing and matrix composition were examined in steroid release from PLGA/PLA NPs by Ishihara et al. [9]. In particular, zinc was capable of interacting with water soluble betamethasone phosphate (BP) to form hydrophobic BP-zinc complexes and improved encapsulation efficiency of BP in NPs. Additionally, bivalent zinc ions formed complexes with PLGA, delaying PLGA degradation and further altering release kinetics of steroid in NPs. The model captures the wide range of release profiles of steroid (Figure 4(f)). In the absence of zinc, PLA NPs release 90% hydrophobic betamethasone dipropionate (BDP) within 5 days. Sustained release of BP was achieved from PLA and PLGA NPs, which were prepared in the presence of zinc ions. If comparing the release of hydrophilic BP to hydrophobic BDP from PLA NPs (Mw 14,000), the simulation shows marked reduction in *k*_*S*_ (5.58 versus 0.099 day^−1^) and Δ*G* (−0.67 versus −6.73 × 10^−21 ^J). Likely, the enhanced hydrophobicity of BP-zinc complexes enables them to strongly interact with PLA NPs. Moreover, the delayed degradation and structural changes of PLA NPs due to the formation of PLA-zinc complexes lower BP diffusivity. In the presence of zinc ions, NPs prepared from PLGA or PLA with a large molecular weight release less BP than those with a low molecular weight, and PLA NPs release less BP than PLGA NPs. Upon increasing the molecular weight of PLGA, the model reveals a decrease in *k*_*off*_ (from 0.336 to 0.042 day^−1^) and Δ*G* (from −1.06 to −1.56 × 10^−21^ J), indicating enhanced BP-PLGA interaction and lowered BP dissociation in NPs formed from PLGA with a large molecular weight. When NPs are prepared from PLA or PLGA with a comparable molecular weight, Δ*G* is smaller in PLA NPs than in PLGA NPs, suggesting that drug-carrier interactions are stronger in PLA NPs than in PLGA NPs. This is consistent with results obtained by Mittal et al. [12]. 

### 3.4. Drug Release from Micro/Nanofibers

Micro/nanofibers with a high surface-to-volume ratio, which can be functionalized by bioactive molecules (e.g., drug, growth factors) [7, 15], may find a wider range of applications in drug delivery and tissue engineering, such as wound healing and tissue regeneration [6, 37, 38]. Like NPs, sustained release from fibers may be achieved through hydrophobic or electrostatic interaction between fibers and encapsulated molecules. For instance, PLLA fibers release hydrophobic doxorubicin base much slower than hydrophilic doxorubicin hydrochloride, due to the enhanced drug-fiber interaction [14]. Likewise, negatively charged heparin may be included in chitosan-alginate fibers, enabling positively charged molecules to form complexes with the fibers [7]. Still, fiber microarchitectures such as pore size also affect the release kinetics of encapsulated molecules [15, 16]. Here, we use the model to analyze the effects of drug hydrophobicity [14], ion pairing [7], fiber microarchitectures [15, 16], and environment temperature [31] on the release kinetics of encapsulated molecules (Figures 5(a)–5(f)). One of these studies reveals limitations of the current model (Figure 5(f)). That is, the model does not consider the erosion and volume change of drug carriers. Parameter estimates for the simulations are listed in Table 3. 

We first analyze the effects of drug hydrophobicity on release kinetics (Figure 5(a)). Zeng et al. [14] encapsulated anticancer drug doxorubicin into electrospun PLLA NFs. Due to its hydrophilicity, doxorubicin hydrochloride could barely be dispersed in a mixture of chloroform and acetone for the electrospinning of PLLA and drug. As a result, a large portion of doxorubicin hydrochloride appeared on the surface of the PLLA NFs. The good solubility of doxorubicin hydrochloride in the release medium led to its rapid release from the PLLA NFs. By adding dilute ammonia, doxorubicin hydrochloride could be converted into lipophilic doxorubicin base, resulting in its uniform distribution in and sustained release from the PLLA NFs. The model captures both the rapid release of doxorubicin hydrochloride and the sustained release of doxorubicin base from the PLLA NFs (Figure 5(a)). Interestingly, doxorubicin hydrochloride and base possess a comparable rate constant of diffusion/convection, *k*_*S*_ (0.027 versus 0.041 min^−1^). However, doxorubicin base displays a much lower Δ*G* than doxorubicin hydrochloride does (−6.65 versus 7.4 × 10^−21 ^J), suggesting that PLLA is capable of retaining and delaying the release of hydrophobic doxorubicin base but not hydrophilic doxorubicin hydrochloride. 

Next, we study the influences of ion pairing on the sustained release of protein from fibers. Liao et al. [7] produced chitosan-alginate fibers from interfacial polyelectrolyte complexation. Heparin, which can interact with many growth factors due to its high negative charge density, has been used for sustained delivery of avidin and PDGF. The model successfully describes the release kinetics of avidin and PDGF (Figures 5(b) and 5(c)). In the absence of heparin, chitosan-alginate fibers release 95% of avidin over a period of 20 days. An addition of heparin into chitosan-alginate fibers not only reduces the initial bust release from 55% to 30%, but also extends the duration of steady release. The effects of heparin concentrations on the release kinetics of avidin and PDGF are captured by the model. Compared to the nonheparin modified fibers, simulation results of the release from the 50 : 50 Ag/HP fibers show reductions in Δ*G* (from 1.03 to about −2.64 × 10^−21 ^J) and *k*_*off*_ (from 0.1 to 0.05 day^−1^), explaining the reduced initial burst and the prolonged steady release. 

PDGF with positive charges electrostatically interacts with the carboxyl groups of alginate, leading to sustained release from chitosan-alginate fibers. Revealed by the model, a negative Δ*G* (−4.1 × 10^−21 ^J) suggests that a small amount of free PDGF is available for the initial burst release (Figure 5(c)). Upon the addition of heparin, Δ*G* is further reduced to −13.5 × 10^−21 ^J. As a result, the sustained release of PDGF is enhanced by including heparin into the fibers. Because heparin is an integrated part of the fibers, PDGF- or avidin-heparin complexes decrease disassociation of proteins from the fibers, leading to a low rate of sustained release (i.e., low *k*_*off*_).

In addition to ion pairing, fiber structure may affect the release kinetics of encapsulated molecules from fibers. Briganti et al. [15] electrospun PEtU-PDMS fibrous scaffolds, which were functionalized in fibrinogen solutions containing heparin and heparin-binding VEGF and bFGF. After the complete polymerization of fibrinogen, fibrin completely covered the PEtU-PDMS fibers, retaining heparin and the growth factors. The concentration of fibrinogen solutions, which were used to treat PEtU-PDMS fibers, influenced the fiber surface morphology and microstructure as well as the subsequent release of the growth factors. When the fibrinogen concentration increased from 10 mg/mL to 20 mg/mL, the release rates of both VEGF and bFGF from the treated fibers decreased greatly. The model is used to illustrate the effects of fibrinogen concentrations and fiber microstructures on the release kinetics of both growth factors (Figure 5(d)). The model reveals reduction in Δ*G*, as a result of an increase in fibrinogen concentration (Table 3). Therefore, changes in the fiber microarchitectures affect the ability of heparin to retain the growth factors. When treated with fibrinogen solutions at the same concentration, the PEtU-PDMS fibers release bFGF slower than VEGF. This is likely due to the different binding capabilities of the growth factors with heparin and fibrin. 

The influences of fiber structure on drug release are also analyzed in another case study (Figure 5(e)). Hong et al. [16] synthesized mesoporous bioactive glass hollow fibers (MBGHFs), which could encapsulate 7 times more drug than solid fibers. Interestingly, long (e.g., 5–10 mm in length) MBGHF fragments released GS much slower than short (2–2.5 mm) fragments. It is believed that the two open ends of a hollow fiber provided another route for drug release in addition to the mesopores. This effect is more pronounced in short MBGHF fragments. Although the model does not explicitly include diffusion through the open ends of hollow fibers, its semiphenomenological nature allows it to capture drug release from hollow fibers. Moreover, the model suggests that shortening fragment length increases the effective rate constant of diffusion/convection *k*_*S*_ (Table 3). This is due to the effects of additional diffusion routes via the ends. Consistently, Δ*G* that measures the strength of drug-fiber interaction also slightly increases. 

Last, we examine the temperature effects on the controlled release of proteins from nanofibers (Figure 5(f)). Loh et al. [31] electrospun thermosensitive poly(PEG/PPG/PCL urethane) hydrogel NFs encapsulating a model protein BSA. BSA release was regulated by adjusting temperature in the range of 25°C to 37°C. When temperature increased, hydrophilic fiber mats expelled water and became hydrophobic. The model suggests both the rate constants of diffusion/convection (*k*_*S*_) and disassociation (*k*_*off*_) increase with temperature (Table 3). Likely, the thermally induced expelling of water enhances the disassociation of and expels BSA from the hydrogels fibers. As a comparison, temperature increase has little effect on BSA release from PCL NFs: *k*_*S*_ and *k*_*off*_ remain the same when temperature increases from 25 to 37°C, while a moderate increase in Δ*G* explains a lightly enhanced burst release. In contrast to the two-phase release of BSA from PCL NFs, hydrogel NFs release BSA in three phases: initial burst release, sustained release, and second burst release. The second burst release of BSA is due to the erosion of hydrogel fibers [31]. The current model captures the first two phases of BSA release from hydrogels fibers, but not the second burst release, because the model does not consider the volume change of drug carriers and its influences on drug release. 

### 3.5. Statistical Analysis for Nonlinear Regression

To validate the model and evaluate the robustness of the parameter determination process, bootstrap sampling is used to study the properties of each model parameter, such as mean and standard deviation. In this process, we assume that the observations in each case are independent. This assumption is satisfied for most cases through testing autocorrelation between observations. Using this method, all the 60 cases are studied, except a few cases (e.g., Figures 3(b), 3(c), 3(e), and 4(a)) whose sample sizes are too small. 

Results from the statistical analysis show that all parameters are significant. Parameter estimates for two selected cases are presented in Tables 4 and 5. At the significant level of 0.05, small *P* values of the *F*-statistic show that the nonlinear model of (4) is significantly different from a simple linear model. Additionally, small *P* values (<0.05) from the bootstrap results show that all the parameters in (4) are significant and should be kept. Nevertheless, the comparable results between bootstrap method and our parameter estimates in Tables 1, 2, and 3 suggest that the nonlinear model is very robust. 

## 4. Conclusion

We evaluated the ability of a simple, three-parameter model to capture the release of bioactive molecules from various nanocarriers. Specifically, the model considers reversible drug-carrier interaction, leading to a closed-form analytical solution. A parameter study illustrated the dependence of release kinetics on each model parameter. Notably, the model resembles 60 sets of release data, which cover a wide spectrum of release kinetics. The model may further our understanding of the underlying mechanisms of sustained release in various delivery systems. Although limitations exist, this model provides a useful tool for the design and synthesis of new nanostructured delivery vesicles, including NPs, nanocapsules, nanofibers, and hollow nanofibers.

## Supplementary Material

A detailed procedure to obtain the analytical solution to the release model was provided in supplementary material. A general procedure was also established to determine the three model parameters, ∆G, k_S_, and k_off_. In addition, the model fit to telmisartan release from mesoporous silica nanoparticle as shown in Figure S1.

## Figures and Tables

**Table 1:** Parameter estimates for simulations in Figure 3.

Drug/Carrier^a^	Mechanisms	Variables	Model parameters^b^		
			*k*_*S*_	*k*_*off*_	Δ*G* (10^−21^ J)
		1 mol% PEG	0.494^M^	0.014^M^	−0.97
CFTSL [24]	PEG addition	3 mol% PEG	1.568^M^	0.026^M^	0.83
		4 mol% PEG	1.273^M^	0.039^M^	2.18
		10 mol% PEG	2.785^M^	0.089^M^	4.77)

		Vera-FBS	2.268^H^	0.092^H^	4.71
Vera and Dox liposome [25]	Serum	Vera-HBS	1.912^H^	0.096^H^	3.88
		Dox-FBS	1.670^H^	0.0037^H^	−10.4
		Dox-HBS	1.706^H^	0.0056^H^	−12.6
		Vera-in vivo	4.561^H^	0.08^H^	11.5
	Lipid membrane	Vera-MLV	3.360^H^	0.04^H^	10.2
		Dox-in vivo	0.212^H^	0.008^H^	−1.22
		Dox-MLV	1.387^H^	0.005^H^	−2.32)

		pH 2.0	0.028^H^	—	—
AmiodaroneLNC [26]	pH	pH 3.0	0.032^H^	0.011^H^	4.52
		pH 4.0	0.033^H^	0.006^H^	3.49
		pH 5.5	0.033^H^	0.004^H^	−0.86
		pH 7.4	0.265^H^	0.003^H^	−9.35)

BSAPLLA [29]	Molecular weight	16,000	0.719^H^	0.025^H^	0.41
		51,000	0.754^H^	0.02^H^	−3.3)

IndomethacinPECL [27]	Coating	PLL-coated	0.0182^M^	0.202^M^	11.8
		uncoated	0.0156^M^	0.203^M^	11.8

^a^CF: carboxylfluorescein; TSL: thermosensitive liposome; Vera: verapamil; Dox: doxorubicin; FBS: fetal bovine serum; HBS: Hepes buffered saline; BSA: bovine serum albumin; PLLA: poly(L-lactide); PECL: poly-*ε*-caprolactone.

^b^ *k*_*on*_ and *k*_*off*_ have different units in difference cases: M: minute^−1^ and H: hour^−1^.

**Table 2:** Parameter estimates for simulations in Figure 4.

Drug/Carrier^a^	Mechanisms	Variables	Model parameters^b^		
			*k*_*S*_	*k*_*off*_	Δ*G* (10^−21^J)
	Pore size	12.9 nm	0.192^M^	—	—
Telmisartan MSNP [30]		7.8 nm	0.1^M^	—	—
		3.6 nm	0.0652^M^	—	—

	Amine additive	no additive	3.913^D^	0.013^D^	5.11
Retinoid Am80PEG-PBLA [11]		TPA	7.314^D^	0.067^D^	6.84
		DMDA	1.272^D^	0.059^D^	6.61
		DMOA	0.541^D^	0.001^D^	−1.18

	Medium ion strength	0.15 M NaCl	0.381^H^	0.023^H^	3.36
DSPLNP [10]		15 mM NaCl	0.248^H^	0.001^H^	4.90
		0.5 mM NaCl	0.258^H^	0.012^H^	0.64
		DDI water	0.237^H^	0.005^H^	−5.1

	Copolymer composition	PLA/PGA 50 : 50	0.197^D^	0.019^D^	−1.69
Estradiol PLGA [12]		PLA/PGA 65 : 35	0.163^D^	0.004^D^	−2.40
		PLA/PGA 85 : 15	0.179^D^	0.007^D^	−3.94

Savoxepine PLA [13]	Particle size	303 nm	1.437^D^	0.064^D^	−0.61
		671 nm	1.792^D^	0.028^D^	−5.52

	Composition and molecular weight	PLGA 8,000	0.333^D^	0.336^D^	−1.06
Steroids PLA/PLGA [9]		PLGA 13,000	0.375^D^	0.04^D^	−1.56
		PLA 9,000	0.319^D^	0.004^D^	−3.2
		PLA 14,000	0.099^D^	0.128^D^	−6.73
	Control (BDP)	PLA 14,000	5.576^D^	0.344^D^	−0.67

^a^MSNP: mesoporous silica nanoparticle; PEG-PBLA: polyethylene glycol-poly(benzyl L-aspartate); DMDA: N,N-dimethyldodecylamine; DMOA: N,N-dimethyloctadecylamine; TPA: triphenylamine; DS: dextran sulfate sodium; PLNPs: polymer-lipid hybrid nanoparticles; PLLA: poly(L-lactide).

^b^ *k*_*on*_ and *k*_*off*_ have different units in difference cases: M: minute^−1^, H: hour^−1^, and D: day^−1^.

**Table 3:** Parameter estimates for simulations in Figure 5.

Drug/Carrier^a^	Mechanisms	Variables	Model parameters^b^		
			*k*_*S*_	*k*_*off*_	Δ*G* (10^−21^ J)
DoxorubicinPLLA fibers [14]	Hydrophobicity	dox hydrochloride	0.027^a^	0.001^a^	7.4
		dox base	0.041^a^	0.0004^a^	−6.65
		100 : 0 AG/HP	11.1^c^	0.095^c^	1.03
Avidin CS-AG fibers [7]		80 : 20 AG/HP	11.2^c^	0.064^c^	−2.37
	Heparin addition	90 : 10 AG/HP	5.12^c^	0.053^c^	−2.09
		50 : 50 AG/HP	9.89^c^	0.045^c^	−2.64
PDGF CS-AG fibers [7]		100 : 0 AG/HP	0.677^c^	0.027^c^	−4.14
		90 : 10 AG/HP	0.486^c^	0.001^c^	−13.5
VEGFPEtU-PDMS/fibrin fibers [15]		10 mg/mL fibrin	0.435^c^	0.009^c^	0.187
	Fibrin concentration	20 mg/mL fibrin	0.434^c^	0.017^c^	−1.27
bFGFPEtU-PDMS/fibrin fibers [15]		10 mg/mL fibrin	0.289^c^	0.001^c^	0.65
		20 mg/mL fibrin	0.242^c^	0.017^c^	−1.94
GSMBGHFs [16]	Fiber length	2–2.5 mm	0.444^b^	0.005^b^	7.24
		5–10 mm	0.136^b^	0.012^b^	5.13
BSAhydrogel nanofibers		37°C	4.81^c^	0.027^c^	−4.9
	Temperature	25°C	1.585^c^	0.017^c^	−5.47
BSAPCL fibers [31]		37°C	1.114^c^	0.011^c^	−4.72
		25°C	1.521^c^	0.011^c^	−6.33

^a^PLLA: poly(L-lactide), Dox: doxorubicin, CS: chitosan, AG: alginate, HP: heparin, PDGF: recombinant human platelet derived growth factor, VEGF: vascular endothelial growth factors, bFGF: basic fibroblast growth factors, PEtU-PDMS: poly(ether)urethane-polydimethylsiloxane, GS: gentamicin sulfate, MBGHFs: mesoporous bioactive glass hollow fibers, BSA: bovine serum albumin, hydrogel fibers: poly(PEG/PPG/PCL urethane) hydrogel fibers, PEG: poly(ethylene glycol), PPG: poly(propylene glycol), and PCL: poly(*ε*-caprolactone).

^b^ *k*_*on*_ and *k*_*off*_ have different units in different cases: M: minute^−1^, H: hour^−1^, and D: day^−1^.

**Table 4:** Properties of the model parameters for Figure 3(a).

Variables	Parameter	Parameter estimation^a^	Bootstrap results			*F*-statistic (*P* value)
			mean	SD^b^	*P* value	
1 mol% PEG	*k*_*S*_	0.4941	0.5592	0.007	<1.0*E* − 6	164.1721 (*P *< 0.001)
	*k*_*off*_	0.0136	0.0169	3.00*E*-04	<1.0*E* − 6	
	*k*_*on*_	0.0172	0.0252	8.00E-04	<1.0*E* − 6	
	Δ*G*	–0.9676	–1.2145	0.0263	<1.0*E* − 6	

3 mol% PEG	*k*_*S*_	1.568	1.4275	0.0182	<1.0*E* − 6	349.7894 (*P *< 0.001)
	*k*_*off*_	0.0261	0.0277	4.00E-04	<1.0*E* − 6	
	*k*_*on*_	0.0213	0.0228	5.00E-04	<1.0*E* − 6	
	Δ*G*	0.8283	0.9656	0.0219	<1.0*E* − 6	

4 mol% PEG	*k*_*S*_	1.2728	1.2165	0.0107	<1.0*E* − 6	470.3850 (*P *< 0.001)
	*k*_*off*_	0.039	0.0418	5.00E-04	<1.0*E* − 6	
	*k*_*on*_	0.023	0.0252	5.00E-04	<1.0*E* − 6	
	Δ*G*	2.1781	2.2303	0.0218	<1.0*E* − 6	

10 mol% PEG	*k*_*S*_	2.7849	2.4222	0.0275	<1.0*E* − 6	782.3148 (*P *< 0.0001)
	*k*_*off*_	0.0891	0.0841	7.00E-04	<1.0*E* − 6	
	*k*_*on*_	0.028	0.0255	3.00E-04	<1.0*E* − 6	
	Δ*G*	4.7684	5.0285	0.0251	<1.0*E* − 6	

^a^Parameters are determined using Matlab codes as detailed in Section 3.1.

^b^SD: standard deviation.

**Table 5:** Properties of the model parameters for Figure 4(d).

Variables	Parameter	Parameter estimation^a^	Bootstrap results			F-statistic (*P* value)
			mean	SD^b^	*P* value	
PLGA50/50	*k*_*S*_	0.1972	0.2035	0.001	<1.0*E* − 6	16.8532 (*P *< 0.004)
	*k*_*off*_	0.0199	0.0203	1.00E-04	<1.0*E* − 6	
	*k*_*on*_	0.03	0.0314	2.00E-04	<1.0*E* − 6	
	Δ*G*	−1.6872	−1.7692	0.0152	<1.0*E* − 6	

PLGA65/35	*k*_*S*_	0.1633	0.1668	0.0032	<1.0*E* − 6	8.4857 (*P *< 0.01)
	*k*_*off*_	0.0044	0.0044	1.00E-04	<1.0*E* − 6	
	*k*_*on*_	0.0079	0.008	2.00E-04	<1.0*E* − 6	
	Δ*G*	−2.3984	−2.4088	0.01	<1.0*E* − 6	

PLGA85/15	*k*_*S*_	0.1795	0.1854	0.0038	<1.0*E* − 6	174.3171 (*P *< 0.001)
	*k*_*off*_	0.0068	0.0068	1.00E-04	<1.0*E* − 6	
	*k*_*on*_	0.0176	0.0181	4.00E-04	<1.0*E* − 6	
	Δ*c*	−3.9408	−3.9607	0.0151	<1.0*E* − 6	

^a^Parameters are determined using a Matlab code as detailed in Section 3.1.

^b^SD: standard deviation.

## References

[1] Torchilin VP (2006). Multifunctional nanocarriers. *Advanced Drug Delivery Reviews*.

[2] Sill TJ, von Recum HA (2008). Electrospinning: applications in drug delivery and tissue engineering. *Biomaterials*.

[3] Kreuter J (1991). Nanoparticle-based drug delivery systems. *Journal of Controlled Release*.

[4] Barenholz Y (2001). Liposome application: problems and prospects. *Current Opinion in Colloid and Interface Science*.

[5] Lian T, Ho RJY (2001). Trends and developments in liposome drug delivery systems. *Journal of Pharmaceutical Sciences*.

[6] Verreck G, Chun I, Rosenblatt J (2003). Incorporation of drugs in an amorphous state into electrospun nanofibers composed of a water-insoluble, nonbiodegradable polymer. *Journal of Controlled Release*.

[7] Liao IC, Wan ACA, Yim EKF, Leong KW (2005). Controlled release from fibers of polyelectrolyte complexes. *Journal of Controlled Release*.

[8] Ye M, Kim S, Park K (2010). Issues in long-term protein delivery using biodegradable microparticles. *Journal of Controlled Release*.

[9] Ishihara T, Izumo N, Higaki M (2005). Role of zinc in formulation of PLGA/PLA nanoparticles encapsulating betamethasone phosphate and its release profile. *Journal of Controlled Release*.

[10] Li Y, Wong HL, Shuhendler AJ, Rauth AM, Wu XY (2008). Molecular interactions, internal structure and drug release kinetics of rationally developed polymer-lipid hybrid nanoparticles. *Journal of Controlled Release*.

[11] Satoh T, Higuchi Y, Kawakami S (2009). Encapsulation of the synthetic retinoids Am80 and LE540 into polymeric micelles and the retinoids’ release control. *Journal of Controlled Release*.

[12] Mittal G, Sahana DK, Bhardwaj V, Ravi Kumar MNV (2007). Estradiol loaded PLGA nanoparticles for oral administration: effect of polymer molecular weight and copolymer composition on release behavior in vitro and in vivo. *Journal of Controlled Release*.

[13] Leroux JC, Allémann E, De Jaeghere F, Doelker E, Gurny R (1996). Biodegradable nanoparticles—from sustained release formulations to improved site specific drug delivery. *Journal of Controlled Release*.

[14] Zeng J, Yang L, Liang Q (2005). Influence of the drug compatibility with polymer solution on the release kinetics of electrospun fiber formulation. *Journal of Controlled Release*.

[15] Briganti E, Spiller D, Mirtelli C (2010). A composite fibrin-based scaffold for controlled delivery of bioactive pro-angiogenetic growth factors. *Journal of Controlled Release*.

[16] Hong Y, Chen X, Jing X, Fan H, Gu Z, Zhang X (2010). Fabrication and drug delivery of ultrathin mesoporous bioactive glass hollow fibers. *Advanced Functional Materials*.

[17] Zolnik BS, Burgess DJ (2008). Evaluation of in vivo-in vitro release of dexamethasone from PLGA microspheres. *Journal of Controlled Release*.

[18] Siepmann J, Siepmann F (2008). Mathematical modeling of drug delivery. *International Journal of Pharmaceutics*.

[19] Enden G, Schroeder A (2009). A mathematical model of drug release from liposomes by low frequency ultrasound. *Annals of Biomedical Engineering*.

[20] Zeng L, Wu X (2010). Modeling the sustained release of lipophilic drugs from liposomes. *Applied Physics Letters*.

[21] Crank J (1975). *The Mathematics of Diffusion*.

[22] Borghi A, Foa E, Balossino R, Migliavacca F, Dubini G (2008). Modelling drug elution from stents: effects of reversible binding in the vascular wall and degradable polymeric matrix. *Computer Methods in Biomechanics and Biomedical Engineering*.

[23] Sakharov DV, Kalachev LV, Rijken DC (2002). Numerical simulation of local pharmacokinetics of a drug after intravascular delivery with an eluting stent. *Journal of Drug Targeting*.

[24] Li L, ten Hagen TLM, Schipper D (2010). Triggered content release from optimized stealth thermosensitive liposomes using mild hyperthermia. *Journal of Controlled Release*.

[25] Shabbits JA, Chiu GNC, Mayer LD (2002). Development of an in vitro drug release assay that accurately predicts in vivo drug retention for liposome-based delivery systems. *Journal of Controlled Release*.

[26] Lamprecht A, Bouligand Y, Benoit JP (2002). New lipid nanocapsules exhibit sustained release properties for amiodarone. *Journal of Controlled Release*.

[27] Calvo P, Vila-Jato JL, Alonso MJ (1997). Evaluation of cationic polymer-coated nanocapsules as ocular drug carriers. *International Journal of Pharmaceutics*.

[28] Efron B, Tibshirani R (1993). *An Introduction to the Bootstrap*.

[29] Lu Z, Bei J, Wang S (1999). A method for the preparation of polymeric nanocapsules without stabilizer. *Journal of Controlled Release*.

[30] Zhang Y, Zhi Z, Jiang T, Zhang J, Wang Z, Wang S (2010). Spherical mesoporous silica nanoparticles for loading and release of the poorly water-soluble drug telmisartan. *Journal of Controlled Release*.

[31] Loh XJ, Peh P, Liao S, Sng C, Li J (2010). Controlled drug release from biodegradable thermoresponsive physical hydrogel nanofibers. *Journal of Controlled Release*.

[32] Mayer LD, Tai LCL, Ko DSC (1989). Influence of vesicle size, lipid composition, and drug-to-lipid ratio on the biological activity of liposomal doxorubicin in mice. *Cancer Research*.

[33] Takeuchi H, Kojima H, Yamamoto H, Kawashima Y (2001). Evaluation of circulation profiles of liposomes coated with hydrophilic polymers having different molecular weights in rats. *Journal of Controlled Release*.

[34] Allen TM, Mehra T, Hansen C, Chin YC (1992). Stealth liposomes: an improved sustained release system for 1-*β*-D- arabinofuranosylcytosine. *Cancer Research*.

[35] Roux E, Passirani C, Scheffold S, Benoit JP, Leroux JC (2004). Serum-stable and long-circulating, PEGylated, pH-sensitive liposomes. *Journal of Controlled Release*.

[36] Yuan F, Dellian M, Fukumura D (1995). Vascular permeability in a human tumor xenograft: molecular size dependence and cutoff size. *Cancer Research*.

[37] Yoshimoto H, Shin YM, Terai H, Vacanti JP (2003). A biodegradable nanofiber scaffold by electrospinning and its potential for bone tissue engineering. *Biomaterials*.

[38] Pham QP, Sharma U, Mikos AG (2006). Electrospinning of polymeric nanofibers for tissue engineering applications: a review. *Tissue Engineering*.

