# Supplementary material for: Modeling Drug-Carrier Interaction in the Drug Release from Nanocarriers
Source: J Drug Deliv. 2011 Aug 10;2011:370308. doi: 10.1155/2011/370308 (PMC3154485; doi:10.1155/2011/370308)
Supplement: Supplementary file 1 — A detailed procedure to obtain the analytical solution to the release model was provided in supplementary material. A general procedure was also established to determine the three model parameters, ∆G, kS, and koff. In addition, the model fit to telmisartan release from mesoporous silica nanoparticle as shown in Figure S1. [file 370308.f1.pdf]

Modeling Drug-Carrier Interaction in the Drug Release from Nanocarriers

Like Zeng[a], *Lingling An*[b], *Xiaoyi Wu*[a,c,*]

[a]Department of Aerospace and Mechanical Engineering, University of Arizona, Tucson, Arizona 85721, USA

[b]Department of Agricultural and Biosystems Engineering, University of Arizona, Tucson, Arizona 85721, USA

[c]Biomedical Engineering IDP and Bio5 Institute, University of Arizona, Tucson, Arizona 85721, USA

[*]Corresponding Author. 1130 N. Mountain Ave., Tucson, Arizona 85721, USA. Tel.: 1-00-520-626-5854. Fax: 1-00-520-621-8198. E-mail: xwu@email.arizona.edu.

Supporting Information

1. A detailed procedure to obtain the solution to the linear system

A detailed procedure to obtain the solution to the linear system of the first-order differential equations (2) and (3) is provided.

$$\frac{dc_\mathrm{F}}{dt} = -(k_\mathrm{S} + k_\mathrm{on})c_\mathrm{F} + k_\mathrm{off}c_\mathrm{A}\ , \tag{2}$$

$$\frac{dc_\mathrm{A}}{dt} = k_\mathrm{on}c_\mathrm{F} - k_\mathrm{off}c_\mathrm{A}\ , \tag{3}$$

First, we normalize c_F and c_A using c_0, and rewrite equations (2) and (3) in the matrix form,

$$\frac{dC}{dt} = -A \cdot C \tag{S1}$$

where

$$\boldsymbol{C} = \begin{pmatrix} c_{\mathrm{F}}/c_0 \\ c_{\mathrm{A}}/c_0 \end{pmatrix}, \qquad \boldsymbol{A} = \begin{pmatrix} k_{\mathrm{S}} + k_{\mathrm{on}} & -k_{\mathrm{off}} \\ -k_{\mathrm{on}} & k_{\mathrm{off}} \end{pmatrix}$$

Assuming $\boldsymbol{C} = \boldsymbol{b}e^{-\lambda t}$, where $\boldsymbol{b}$ is the coefficient vector, equation (S1) is reduced to

$$(\boldsymbol{A} - \lambda \boldsymbol{I}) \cdot \boldsymbol{b} = 0 \tag{S2}$$

Clearly, λ and $\boldsymbol{b}$ are the eigenvalue and eigenvector of $\boldsymbol{A}$, respectively. The characteristic equation of the eigenvalue problem is

$$(k_{\mathrm{S}} + k_{\mathrm{on}} - \lambda)(k_{\mathrm{off}} - \lambda) - k_{\mathrm{on}}k_{\mathrm{off}} = 0\ , \tag{S3}$$

or

$$\lambda^2 - (k_{\mathrm{S}} + k_{\mathrm{on}} + k_{\mathrm{off}})\lambda + k_{\mathrm{S}}k_{\mathrm{off}} = 0\ . \tag{S4}$$

By solving equation (S4), one may obtain λ_1 and λ_2 as follows

$$\lambda_{1,2} = \left[k_{\mathrm{S}} + k_{\mathrm{on}} + k_{\mathrm{off}} \pm \sqrt{(k_{\mathrm{S}} + k_{\mathrm{on}} + k_{\mathrm{off}})^2 - 4k_{\mathrm{S}}k_{\mathrm{off}}}\right]/2. \tag{S5}$$

Using equation (S2), the eigenvectors corresponding to λ_1 and λ_2 can be obtained

$$\boldsymbol{b}_1 = \alpha_1 \begin{pmatrix} k_{\mathrm{off}} \\ k_{\mathrm{S}} + k_{\mathrm{on}} - \lambda_1 \end{pmatrix}, \boldsymbol{b}_1 = \alpha_2 \begin{pmatrix} k_{\mathrm{off}} \\ k_{\mathrm{S}} + k_{\mathrm{on}} - \lambda_2 \end{pmatrix} \tag{S6}$$

Here α_1 and α_2 are two non-zero coefficients. Accordingly, the solution to equation (S1) is

$$\boldsymbol{C} = \boldsymbol{b}_1 e^{-\lambda_1 t} + \boldsymbol{b}_2 e^{-\lambda_2 t} \tag{S7}$$

Equivalently,

$$c_{\mathrm{F}}/c_0 = \alpha_1 k_{\mathrm{off}} e^{-\lambda_1 t} + \alpha_2 k_{\mathrm{off}} e^{-\lambda_2 t} \tag{S8}$$

$$c_{\mathrm{A}}/c_0 = \alpha_1 (k_{\mathrm{S}} + k_{\mathrm{on}} - \lambda_1) e^{-\lambda_1 t} + \alpha_2 (k_{\mathrm{S}} + k_{\mathrm{on}} - \lambda_2) e^{-\lambda_2 t} \tag{S9}$$

Using the initial conditions $c_{\mathrm{F}}(0)/c_0 = k_{\mathrm{off}}/(k_{\mathrm{on}}+k_{\mathrm{off}})$ and $c_{\mathrm{A}}(0)/c_0 = k_{\mathrm{on}}/(k_{\mathrm{on}}+k_{\mathrm{off}})$, α_1 and α_2 are determined to be

$$\alpha_1 = \frac{k_S - \lambda_2}{(k_{on} + k_{off})(\lambda_1 - \lambda_2)} \quad \text{(S10)}$$

$$\alpha_2 = \frac{\lambda_1 - k_S}{(k_{on} + k_{off})(\lambda_1 - \lambda_2)} \quad \text{(S11)}$$

Bringing α_1 and α_2 into equations (S8) and (S9), one may obtain the solution provided in the main text

$$\frac{c_F(t)}{c_0} = \frac{k_{off}}{k_{on} + k_{off}} \left[\frac{k_S - \lambda_2}{\lambda_1 - \lambda_2} e^{-\lambda_1 t} + \frac{\lambda_1 - k_S}{\lambda_1 - \lambda_2} e^{-\lambda_2 t} \right] \quad (4)$$

$$\frac{c_A(t)}{c_0} = \frac{k_{on}}{k_{on} + k_{off}} \left[\frac{-\lambda_2}{\lambda_1 - \lambda_2} e^{-\lambda_1 t} + \frac{\lambda_1}{\lambda_1 - \lambda_2} e^{-\lambda_2 t} \right]. \quad (5)$$

2. A detailed procedure to determine model parameters

We have developed a simple procedure to estimate each model parameter, which is further optimized using a customer code in Matlab 7.0. Fig. 3A is used as an example to illustrate this process.

2.1. Estimate ΔG

We begin with estimating ΔG. The magnitude of the initial release can be approximated using Eq. 8,

$$\frac{k_{off}}{k_{on} + k_{off}} = \frac{1}{1 + exp(-\Delta G / k_{BT})} \quad \text{(S12)}$$

In Fig.3A, The magnitude of the initial release is estimated to be 0.45, leading to

$$\frac{1}{1 + exp(-\Delta G / k_{BT})} = 0.45 \quad \text{(S13)}$$

As a result, ΔG = -0.83.

2.2. Estimate k_S

When *t* approaches to 0, Eq.6 in the main text can be expanded using Taylor expansion. Truncating the high-order terms, Eq.6 is reduced to,

$$\frac{M_t}{M_0} = \frac{\lambda_1 \lambda_2}{k_{on} + k_{off}} t = \frac{k_S k_{off}}{k_{on} + k_{off}} t \quad \text{(S14)}$$

Substituting Eq. S12 into Eq. S14 yields:

$$\frac{M_t}{M_0} = \frac{k_S k_{\text{off}}}{k_{\text{on}}+k_{\text{off}}} t = \frac{k_S}{1+exp(-\Delta G/k_B T)} t \qquad \text{(S15)}$$

Eq. S16 suggests that the initial release can be approximated by a linear function. Moreover, the release rate that is equivalent to the slope of the linear function is $k_S/\left(1 + exp(-\Delta G/k_B\,T)\right)$. Noting Eq. S16 is valid at small *t*. In Fig. 3A, the release rate is estimated to be 0.2 at $t \approx 0$, resulting in,

$$k_S = 0.2\left(1 + exp(-\Delta G/k_B T)\right) \qquad \text{(S16)}$$

We thus obtain $k_S = 0.44$.

2.3.Estimate k_{off}

We estimate k_{off} by rearranging Eq.6

$$ln\left(1 - \frac{M_t}{M}\right) = -ln\left(\frac{\lambda_2(k_S-\lambda_2)}{(k_{\text{on}}+k_{\text{off}})(\lambda_1-\lambda_2)} e^{-\lambda_1 t} + \frac{\lambda_1(\lambda_1-k_S)}{(k_{\text{on}}+k_{\text{off}})(\lambda_1-\lambda_2)} e^{-\lambda_2 t}\right) \qquad \text{(S17)}$$

The first term on the right-hand side of Eq. S17 represents the residual of the initial burst release, and the second term represents the residual of the sustained release. When t is large enough, the residual of the initial burst release can be neglected. Then, taking natural logarithm on the both sides of Eq.S17, one may have

$$ln\left(1 - \frac{M_t}{M}\right) = ln\left(\frac{\lambda_1(\lambda_1-k_S)}{(k_{\text{on}}+k_{\text{off}})(\lambda_1-\lambda_2)}\right) -\lambda_2 t \qquad \text{(S18)}$$

Eq. S18 provides a mean to estimate λ_2 using the slope of $ln(1 - M_t/M_0)$. In Fig. 3A, the slope of $ln(1 - M_t/M_0)$ that equals to λ_2 is estimated to be 0094. Bringing it into Eq. S5, we obtained k_{off} = 0.01.

Therefore, the three model parameters are estimated to be $k_S = 0.44$, $k_{\text{off}} = 0.01$, and $\Delta G = -0.83$, respectively. Using these estimations, a Matlab optimization returns $k_S = 0.494$, $k_{\text{off}} = 0.0136$, and $\Delta G = -0.968$.

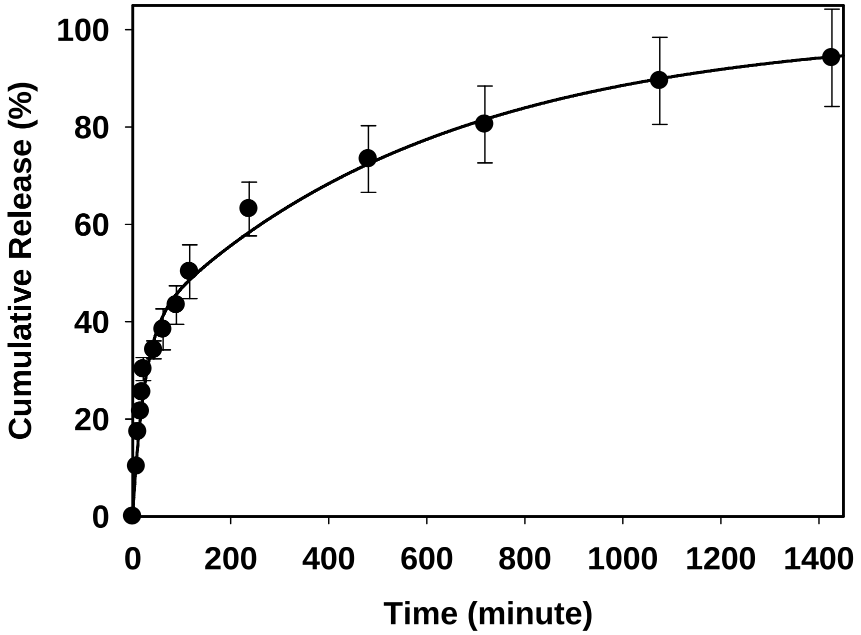

Fig. S1. The mode fit to telmisartan (TEL) release from mesoporous silica nanoparticle (MSNP). Solid line represents the model prediction. Parameters used for the simulation are: k_S = 0.04 minute^{-1}, k_{off} = 0.018 minute^{-1}, and $\Delta G = -1.2 \times 10^{-21}$J.
